# Association between wine consumption and migraine: a systematic review and meta-analysis of cross-sectional

**DOI:** 10.1093/alcalc/agaf004

**Published:** 2025-02-14

**Authors:** Maribel Lucerón-Lucas-Torres, Marta C Ruiz-Grao, Carlos Pascual-Morena, Susana Priego-Jiménez, María López-González, Celia Álvarez-Bueno

**Affiliations:** Centro de Estudios Socio-Sanitarios, Grupo de Investigación Age-ABC, Universidad de Castilla-La Mancha, Cuenca, 16002, Spain; Department of Nursing, University of Castilla-La Mancha, Campus Universitario s/n - 02071 - Albacete, Spain; Centro de Estudios Socio-Sanitarios, Grupo de Investigación Age-ABC, Universidad de Castilla-La Mancha, Cuenca, 16002, Spain; Department of Nursing, University of Castilla-La Mancha, Campus Universitario s/n - 02071 - Albacete, Spain; Department of Nursing, University of Castilla-La Mancha, Campus Universitario s/n - 02071 - Albacete, Spain; Centro de Estudios Socio-Sanitarios, Grupo de Investigación Age-ABC, Universidad de Castilla-La Mancha, Cuenca, 16002, Spain; Hospital Virgen de la Luz, C/Hermandad Donantes de Sangre, 1, 16002, Cuenca, Spain; Centro de Estudios Socio-Sanitarios, Grupo de Investigación Age-ABC, Universidad de Castilla-La Mancha, Cuenca, 16002, Spain; Centro de Estudios Socio-Sanitarios, Grupo de Investigación Age-ABC, Universidad de Castilla-La Mancha, Cuenca, 16002, Spain; Facultad de Ciencias de la Salud, Universidad Autónoma de Chile, Talca, Chile

**Keywords:** wine consumption, alcohol, migraine, adults

## Abstract

**Background:**

It seems that diet is one of the main triggers of migraine; one of the most studied is alcohol, and also, over the years, red wine has been shown to trigger headaches. Therefore, this systematic review and meta-analysis aims to examine the strength of the association between wine consumption and migraine.

**Methods:**

In this systematic review and meta-analysis, a search of MEDLINE (via PubMed), Scopus, Cochrane, and Web of Science databases was conducted to assess the association between wine consumption and migraine, covering baseline to December 2023. Pooled Odds Ratio (p-OR) were calculated using the DerSimonian and Laird methods. This study was previously registered in PROSPERO (CRD42024511115). The risk of bias was evaluated using The Quality Assessment Tool for Observational Cohort and Cross-Sectional Studies.

**Results:**

Five studies were included in this systematic review, and only four of them were in the meta-analysis. Using the DerSimonian and Laird method, the p-OR for the effect of wine consumption on migraine was 0.63 (95% CI 0.36–1.09). The included studies after the risk of bias assessment showed a moderate risk of bias.

**Conclusions:**

The findings of this systematic review and meta-analysis indicate that there is no conclusive evidence to support an increased or decreased risk of migraine associated with wine consumption.

## Introduction

Migraine is a neurological disorder characterized by recurrent episodes of headache accompanied by changes in the nervous system, gastrointestinal tract, and sensory functions (Ashina et al. 2021, Waliszewska-Prosół et al. 2021, Ferrari et al. 2022, Raggi et al. 2024), as the symptoms apart from headache are nausea, vomiting, phonophobia, and/or photophobia (Pescador Ruschel and De Jesus 2022). Migraine is one of the most disabling diseases in the world. Migraine is classified as moderate to severe in intensity and can often be unilateral (Waliszewska-Prosół et al. 2024). The prevalence of migraine is between 15% and 20% and accounts for almost 6% of the global burden of disease (GBD 2019). Although these estimates do not represent the real world, there are different scales or tools to measure the disability produced by this pathology, which, although difficult, allow the disability to be objectified (Waliszewska-Prosół et al. 2024). Migraine has a major social and economic impact (MacGregor 2017), as it affects the quality of life of the people who suffer from it, even interrupting their working life (Pescador Ruschel and De Jesus 2022). The concept of migraine has been distinguished from other headaches and has evolved over time, although it is still undergoing refinement and modification today (Goadsby and Evers 2020). This disorder is classified into migraine with aura and migraine without aura (IHS 2018).

Over the years, the possible factors that cause migraine have been studied. Genetic plays an important role in this disorder, or endogenous factors such as menstruation, which are not modifiable, but environmental or exogenous factors such as diet are also important and can be acted upon (Magis and Schoenen 2012). Diet has been shown to be one of the main triggers of migraine, and has been the subject of study for a long time and continues to be so today because patients with migraine have reported that certain foods trigger migraine attacks (Pavlovic et al. 2014, Hindiyeh et al. 2020). Some of the foods reported to trigger migraine attacks are chocolate, caffeine, milk, cheese, fatty foods, citrus fruits, and alcohol (Fukui et al. 2008, Rockett et al. 2012, Hoffmann and Recober 2013). A systematic review found that fasting and alcohol consumption are migraine triggers in 44% and 27% of cases, respectively (Peroutka 2014).

There is currently a debate about whether alcohol aggravates migraine or not. Excessive alcohol consumption can cause headaches, commonly known as hangovers (Panconesi 2008). However, wine-induced headaches can arise even with moderate amounts, usually within 30 minutes to three hours after consumption (Panconesi 2008). Red wine has traditionally been shown to cause headaches even in nonmigraine sufferers (Littlewood et al. 1988, Panconesi 2008). An earlier study indicated that low amounts of alcohol consumption did not cause headache pain, suggesting that its role as a migraine trigger may have been exaggerated (Vives-Mestres et al. 2022). However, other reports suggest that both alcohol and red or white wine can trigger migraines (Fukui et al. 2008). Due to the unclear relationship between wine intake and the development of migraine, this systematic review and meta-analysis aims to examine the strength of the association between wine consumption and migraine.

## Methods

### Search strategy and study selection

The Preferred Reporting Items for Systematic Reviews and Meta-Analyses (PRISMA) (Page et al. 2021) guidelines were followed to report this systematic review and meta-analysis and the recommendations of the Cochrane Collaboration Handbook were followed in conducting it (Higgins et al. 2024). The meta-analysis and systematic review were registered on PROSPERO under the registration number CRD42024511115.

From the start until 7 February 2024, an exhaustive search was carried out in the MEDLINE (via PubMed), Scopus, Cochrane, and Web of Science databases. The search strategy included the following free terms: (i) “migraine,” “headache,” (ii) “alcohol,” “wine,” and “alcohol consumption” and (iii) “adults,” “young adults,” “older,” “elderly.” Finally, relevant research was found by looking through the reference lists of the papers that were part of this systematic review. The complete search strategy for MEDLINE is shown in Table S1.

### Eligibility

Articles considered for inclusion included cross-sectional studies assessing the association between wine consumption and migraine. Two independent reviewers (MLLT and CAB) conducted the search and selection of these studies, analyzing titles and abstracts. Inclusion criteria were as follows: (i) patients: general population; (ii) outcome: migraine; (iii) study design: cross-sectional studies; and (iv) studies reporting wine consumption. Studies were excluded when (i) consisted of literature reviews, editorials, or patient case reports. No language restrictions were placed on the search or study selection process. Items were included when the headache being treated in the study is a migraine; we define migraine as a headache of moderate to severe intensity, which is usually associated with various transient symptoms such as sensitivity to light, sound, nausea, vertigo, and dizziness (Dodick 2018).

### Data extraction and quality assessment

The following information was obtained from the studies that were considered for inclusion: (i) study name, (ii) country, (iii) study design, (iv) characteristics of the participants (sample size and percentage by gender, mean age, and type of population (including whether it is a particular population group, study subjects, or population with a pathology)), (v) type of wine (red or white wine)/quantity of wine, and (vi) outcome assessment.

The Quality Assessment Tool for Observational Cohort and Cross-Sectional Studies from the United States National Institute of Health National Heart, Lung, and Blood Institute was used to assess the risk of bias (NHLBI 2021). This tool examines 14 items from which only 12 are applied in cross-sectional studies within the following domains: quality of the research question, reporting of the population definition, participation rate, recruitment, sample size, appropriateness of statistical analyses, timeframe for associations, exposure levels, ascertainment of the exposure, appropriateness of the outcome measured, outcome blinding of researchers, and confounding variables. Each criterion could be scored as “yes” when the study met the criterion or “no” when the study did not meet the criterion. The criteria could also be scored as “not reported” when studies did not clearly report the required information.

### Statistical analysis and data synthesis

A meta-analysis was conducted to determine how wine consumption and migraine prevalence were associated. The meta-analysis included the odds ratio (OR) for the association between wine drinking and migraine. The pooled estimate of the odds ratio (p-OR) and their corresponding 95% confidence intervals (95% CIs) were calculated using the DerSimonian and Lair method, as well as random effects approaches (DerSimonian and Kacker 2007). The I2 statistic was employed to assess the level of heterogeneity, varying between 0% and 100% (Higgins 2002). According to the I2 values, inconsistency was categorized as not important (0%–40%), moderate (30%–60%), substantial (50%–90%), or considerable (75%–100%). The corresponding p-values were also considered (Stettler et al. 2008).

To evaluate the robustness of the summary estimates, sensitivity analyses were performed by systematically excluding each study one at a time from the pooled estimations. Sensitivity analysis allows the impact of individual studies on the overall results to be assessed (Tian 2013). This approach is widely recognized and recommended for identifying influential studies and ensuring the robustness of the findings (Tian 2013). Meta-regression analyses were conducted to determine whether continuous variables, such as the mean age and percentage of women, affect the associations between wine consumption and migraine (Tian 2013). Finally, the effect of small studies was assessed via Egger’s test (Sterne et al. 2001), with a *P*-value < 0.10 indicating significant publication bias. The analyses were carried out via Stata 16.0 (Stata, College Station, TX, USA).

Study selection, data extraction, and risk of bias assessments were performed by two independent reviewers (ML-LT and CA-B). Disagreements were resolved by consensus or with the intervention of a third investigator (MCR-G).

## Results

### Study selection

**Figure 1:**
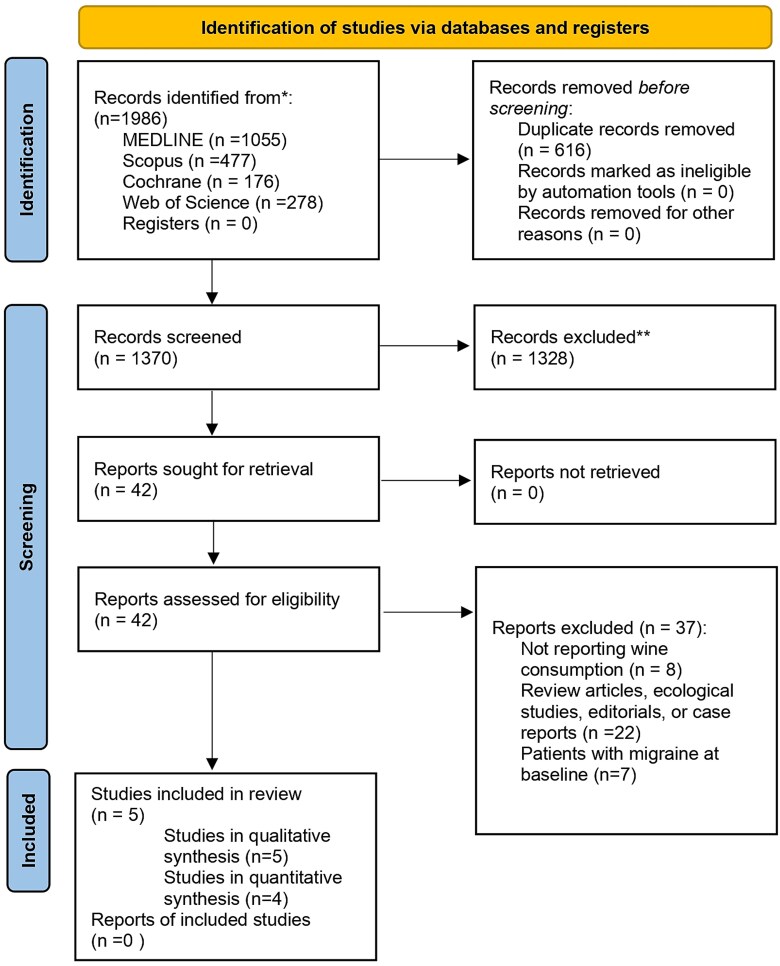
PRISMA 2020 flow diagram for new systematic reviews, which included searches of databases

The search retrieved 1986 articles. 1370 papers were chosen after duplicates were eliminated by looking at the abstract and title; five of these studies (Peatfield 1995, Takeshima et al. 2004, Aamodt et al. 2006, Rist et al. 2015, García-Azorín et al. 2020) were included in this systematic review and only four of these studies (Peatfield 1995, Aamodt et al. 2006, Rist et al. 2015, García-Azorín et al. 2020) were included in the meta-analysis because one of the studies did not provide the OR on wine consumption and migraine, or the data to calculate it (Takeshima et al. 2004) (Fig. 1).

### Study and intervention characteristics

All of the included studies (Peatfield 1995, Takeshima et al. 2004, Aamodt et al. 2006, Rist et al. 2015, García-Azorín et al. 2020) were published between 1995 and 2020, each of them carried out in a different country, including England (Peatfield 1995), Japan (Takeshima et al. 2004), Norway (Aamodt et al. 2006), USA (Rist et al. 2015), and Spain (García-Azorín et al. 2020). The included studies had sample sizes ranging from 527 to 51 383, comprising a total of 65 902 persons aged 20 years or older. All studies included a general population without migraine at baseline.

One of the studies involved patients attending a clinic in that country that were seen by a neurologist who asked them about precipitating factors for migraine (Peatfield 1995). The study carried out in Japan included participants from a rural area and was administered a questionnaire beforehand to find out the health status and lifestyles of the study population, and then they were given different questionnaires about headaches (Takeshima et al. 2004). The Norway study included the entire population aged 20 years and older and administered two questionnaires with more than 200 health-related questions (Aamodt et al. 2006). One of the included studies was conducted in women and also included questionnaires asking about dietary information and headache (Rist et al. 2015). The study carried out in Spain included both students and university professors (García-Azorín et al. 2020) (Table 1).

**Table 1:** Characteristics of the studies included in this systematic review and meta-analysis.

**Reference**	**Country**	**Characteristics of the participants**			**Type of wine/ quantity of wine**	**Participating women of reproductive age**	**Multivariate-adjusted**	**Outcome assessment**
		***N*, women(%)**	**Age (SD or CI)**	**Type of population**				
Peatfield 1995	United Kingdom	527 (NR)	NR	Patients attending the Princess Margaret Migraine Clinic.	Red wine/NR	Age not reported does not report whether women are of reproductive age.	NR	Neurologist
Takeshima et al. 2004	Japan	5758 (53.44)	≥20 (NR)	General population.	General wine/NR	The prevalence of migraine was significantly high in young and middle-aged women.	Age and gender.	Questionnaires
Aamodt et al. 2006	Norway	51,383 (NR)	≥20 (NR)	Nord-Trøndelag Health Study.	General wine/NR	Does not report whether women are of reproductive age.	Sex, age, education, anxiety, depression, coffee consumption, blood pressure, and use of antihypertensives, analgesics or other medications and smoking.	Questionnaires
Rist et al. 2015	USA	7042	53.6 (6.4)	Women’s Health Study.	White wine Red wine/ NR	Women were not of reproductive age.	Age, BMI, exercise, smoking status, average daily calorie consumption, history of diabetes, history of hypertension, treatment for high blood pressure, history of high cholesterol, and treatment for high cholesterol.	Questionnaires
García-Azorín et al. 2020	Spain	1192 (57.7)	22.8 (22,3–23.4)	University and college teachers.	General wine/NR	Does not report whether women are of reproductive age.	NR	Questionnaires

SD, standard deviation. CI, confidence interval. NR, not reported. BMI, body mass index.

Two of the included studies included information on migraine and nonmigraine headaches (Aamodt et al. 2006, Rist et al. 2015). Two studies included information on migraine and tension headaches (Peatfield 1995, Takeshima et al. 2004). Another study classified two headache phenotypes, orthostatic or migraine (García-Azorín et al. 2020). However, in all the included studies only information on migraine headaches was used.

The study not included in the meta-analysis shows that wine is an uncommon trigger for headaches. It is not reported in migraines with aura and affects only 1.4% of migraines without aura. Its impact is minimal and limited to certain cases (Takeshima et al. 2004).

### Risk of bias

The included studies accomplished between seven and ten criteria after the assessment of the risk of bias. All studies provided information on the quality of the research question, population definition, participation rate, recruitment, exposure ascertainment, appropriateness of the measured outcome, and confounding variables. Two studies (40%) reported on sample size justification. Only one study (20%) included information on domains related to exposure levels. The repeated exposure assessment item was measured in only one study. (20%) No study provided information on the blinding of assessors. The domains exposure assessed prior to outcome measurement (question 6) and sufficient timeframe to see an effect (question 7) were always answered ‘no’ because of the cross-sectional design of all studies. (Table S2).

### Meta-analysis, sensitivity analysis, and publication bias

**Figure 2:**
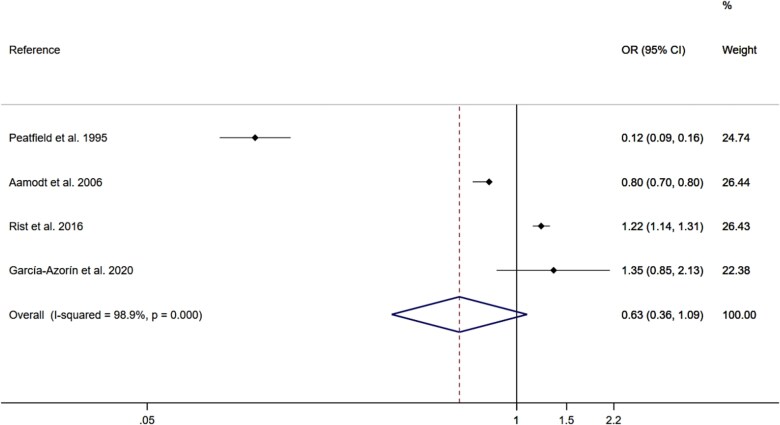
Meta-analysis for the association of wine consumption with migraine

Using the DerSimonian and Lair random effect models, the p-OR for the association of wine consumption with migraine was 0.63 (95% CI 0.36–1.09; I^2^ = 98.9%; τ^2^: 0.2955) (Fig. 2). Pooled OR estimates were not affected after removing one study at a time from the analyses (Table S3). The risk of publication bias could not be assessed via Egger’s test because the number of included studies was <10.

## Discussion

This study is the first to synthesize previous evidence on the association between wine consumption and migraine in the general population. The meta-analysis conducted on the association between wine consumption and migraine revealed no significant findings, suggesting that there is no conclusive evidence to support an increased probability of suffering migraine with wine consumption.

Our data indicates that the consumption of wine is not related to migraines; previous evidence is inconclusive. For decades, wine has been associated as an aggravating factor for the onset of migraine (Littlewood et al. 1988, Sandler et al. 1995, Davis-Martin et al. 2017). There are studies that report consistent evidence that wine was associated with a lower risk of migraine (Liu et al. 2023). It is important to consider the possibility that this apparent protection may be the result of people with migraine tending to consume less alcohol because it may trigger their migraine attacks, thus attributing reverse causality (Evans et al. 2015, Onderwater et al. 2019, Yuan et al. 2022).

Migraines are complex neurological disorders that arise from alterations in sensory networks, causing abnormal processing of both peripheral and central neural signals (Goadsby et al. 2009). This phenomenon involves multiple areas of the brain and is characterized by the activation of nerve pathways between the trigemino-vascular system and the trigemino-cervical complex (Goadsby et al. 2009). In migraine, intense sensory symptoms are transmitted along with the headache, such as sensitivity to light, sound, certain smells, nausea, dizziness, and gastrointestinal discomfort (Goadsby et al. 2009). The intensity of pain suggests a crucial role of pain receptor activation in this disorder (Goadsby et al. 2009). It is common for migraine sufferers to have certain foods that trigger their attacks, although these foods vary from patient to patient. For this reason, specialists suggest that each patient should keep a migraine log to identify which foods may be triggers and avoid them (O’Neal and Murray 2012). Foods that have been most closely linked to migraine triggers include aged cheese, red wine, chocolate, monosodium glutamate, citrus fruits, foods containing nitrates such as sausages or cured meats, and those containing tyramine such as tofu or soy (Crawford et al. 2006, O’Neal and Murray 2012).

The connection between migraine and diet is complex, as both the occurrence of migraines and dietary choices can affect each other (Gazerani 2020). Mendelian randomization (MR) uses genetic variants associated with a risk factor to investigate their causal relationship with health outcomes. Previous MRI-based evidence has supported the idea that certain dietary habits, such as alcohol and coffee consumption, may have a protective effect against migraine (Peroutka 2014). However, uncertainty remains as to whether other dietary habits, such as the intake of different types of bread and milk, exert a causal effect on the development of this condition (Gazerani 2021).

The reason why alcohol may worsen migraine is possibly related to histamine, as it has been known for quite some time to be responsible for vascular headaches (Worm et al. 2019). A deficiency of the enzyme diamine oxidase causes difficulty in the breakdown of histamine, resulting in histamine intolerance (Panconesi 2008). Alcohol can increase the amount of histamine in food by blocking the action of the diamine oxidase enzyme. Red wine tends to have higher levels of histamine compared to white wine (Panconesi 2008). However, although histamines can trigger headaches, it seems unlikely that they are the main cause of migraines associated with wine consumption (Panconesi 2008). Previous research indicates that dietary histamine affects a minority of individuals and that histamine levels are similar in patients who are sensitive and non-sensitive to wine-related migraine triggers (Pergolizzi et al. 2019).

There are different components present in wine that could explain migraines. One of them is sulfites linked to headaches and asthma, commonly used in wine to stop fermentation (Food and drink 2002, Panconesi 2008). Nowadays there are many foods that contain sulfites without producing any type of migraine (Panconesi 2008). White wine has more sulfites than red wine and they are also less associated with migraines, so the theory of migraines produced by this compound loses strength (Panconesi 2008). What is suggested is that they can produce migraine headaches in sensitive individuals when consuming wine since this compound releases histamines in wine, but they are not considered the main cause of migraine headaches (Nicolodi and Sicuteri 1999, Panconesi 2008).

Phenols are also present in wine, which could inhibit the enzymatic activity of PST, increasing the accumulation of tyramine and phenols in the body. Tannins, another component of wine, could be an aggravating factor in migraine, but this theory loses strength because they are found in other foods, such as tea, without being associated with migraine headaches (Nicolodi and Sicuteri 1999). Tyramine, a compound found in cheese, chocolate, and wine, is associated with migraine headaches because they all contain tyramine, but previous evidence has shown that wine is unlikely to produce migraine headache pain from this compound due to its low content (Pattichis et al. 1995).

Red wine is able to trigger the release of serotonin or 5-hydroxytryptamine (5-HT) from platelets and inhibits the reuptake of 5-HT and noradrenaline due to flavonoids and resveratrol present in wine (Panconesi and Sicuteri 1997, Davis-Martin et al. 2017). When 5-HT is released from platelets, it directly or indirectly stimulates the 5-HT2 receptors that would trigger migraine (Panconesi and Sicuteri 1997). But it is suggested that certain elements in wine, such as phenols or polyphenols, may block the action of SULT enzymes, which normally inactivate dopamine in the body, which could lead to an increase in dopamine levels, resulting in migraine headaches in susceptible people. However, it is not yet known which substance, in particular, produces this enzyme inhibition and, therefore, produces the migraine headaches associated with their consumption (Pergolizzi et al. 2019).

The relationship between ethanol and the onset of migraines is still not completely clear, as multiple factors are thought to contribute to migraine triggers. These include the level of individual hypersensitivity and the amount of alcohol consumed (Panconesi et al. 2011). In addition, enzymes involved in alcohol metabolisms, such as alcohol dehydrogenase (ADH) and acetaldehyde dehydrogenase (ALDH), may play a role, especially certain genetic variants of ADH, such as the ADH2 His allele (Ramchandani et al. 2006, García-Martín et al. 2010). Studies have shown that carriers of this allele experience more migraine episodes than noncarriers do, although this hypothesis is still under debate (Ramchandani et al. 2006). On the other hand, mechanisms such as alcohol-induced vasodilation mediated by nitric oxide and the release of calcitonin gene-related peptides in sensory nerve terminals may also be involved in the development of migraines (Schramm et al. 2013). However, this approach seems to be insufficient, and the involvement of receptors in the cortex or brainstem is known (Schramm et al. 2013). A previous meta-analysis reported an inverse correlation between alcohol and migraine, i.e. they reported a lower risk of migraine in people who consumed alcohol (Błaszczyk et al. 2023). However, this study revealed that migraine sufferers consumed less alcohol, suggesting that migraine leads to alcohol avoidance rather than alcohol having a protective role against migraine (Błaszczyk et al. 2023). Our meta-analysis could not demonstrate any association between wine consumption and migraine, so we cannot make any practical implications or specific recommendations on wine consumption in migraine patients. Previous evidence reports that the amount of alcohol consumed could influence the onset or absence of migraine, reporting that very light consumption of 1–2 servings of alcohol is not correlated with this pathology, but exceeding this consumption is associated with an increased risk (Rasmussen 1993, Hirata et al. 2023).

Our study has several limitations that should be mentioned. First, our study included a low number of studies, which may affect the robustness of the results, increasing susceptibility to bias and reducing the generalizability of the findings. Second, there is no common standardized questionnaire for these studies, each assessing wine-producing symptoms differently, making it difficult to compare results directly and, therefore, to synthesize the evidence. Third, it was not possible to analyze the effects of different types of wine and the amounts of wine consumed, as there was insufficient evidence, which is very important, as the specific characteristics of each type of wine, with its alcohol content, polyphenols, and other bioactive compounds, can have different influences on the results. Fourth, a gray literature search was not included, reducing comprehensiveness and affecting the external validation of the conclusions, but it is true that including this type in the literature affects the quality and can lead to problems in the accessibility or synthesis of the review. Fifth, it was not possible to search the Embase database, so some articles of interest may have been missed. Sixth, the risk of bias in the studies was mostly fair, compromising the validity and reliability of the results. Seventh, owing to the lack of data in the included studies, it was not possible to perform meta-regressions, which limits the ability to explore more detailed relationships between variables, and affects the robustness of the conclusions. Eighth, the design of the included studies does not allow a causal relationship to be established, as they do not adequately control for confounding variables and do not establish clear timing between exposure and outcome. Ninth, there are large gaps in our study that would be interesting to address for future research, such as not having been able to carry out analyses owing to the lack of evidence by gender, age, type of alcoholic beverage, consumption, and having little evidence, it would have been interesting to include more articles to have more consistent results that could be extrapolated to the population. Finally, there may be heterogeneity in the populations included in the different studies; therefore, the diversity among study populations may make it difficult to generalize the results, as differences in demographic, cultural, or clinical characteristics may influence the findings.

This systematic review and meta-analysis concluded that there is no conclusive evidence to support an increased probability of suffering migraine associated with wine consumption, as our meta-analysis did not reveal significant results. Taking into account the results of this study, it would be important to continue researching this relationship, distinguishing by type of wine to be able to observe how different types of wine affect migraines depending on their characteristics, as it would be interesting to know if there is any component that is more related to the appearance of this pathology. It would be interesting to distinguish the association of migraine with the dose of wine, to be able to know with what dose a greater number of migraines is produced, and thus conclude if there are differences depending on the quantity and type of wine. Another interesting point for future lines of research is to improve the design of the studies since the studies included in this meta-analysis are cross-sectional, and perhaps other designs could provide higher-quality evidence. In addition, another possible line of research would be to investigate the effects of other types of alcohol separately on the onset of migraine.

## Supplementary Material

Material_suplementario_agaf004

## Data Availability

Corresponding author.

## Notes

Registration ID: CRD42024511115 (PROSPERO)
