# Supplementary material for: Association between wine consumption and migraine: a systematic review and meta-analysis of cross-sectional
Source: Alcohol Alcohol. 2025 Feb 14;60(2):agaf004. doi: 10.1093/alcalc/agaf004 (PMC11826089; doi:10.1093/alcalc/agaf004)
Supplement: Material_suplementario_agaf004 [file material_suplementario_agaf004.docx]

**SUPPLEMENTARY MATERIAL**

**Table S1.** Complete search strategy for MEDLINE.

| **Population** | **Intervention** | **Outcome** |
| --- | --- | --- |
| Adults  OR  Young adults  OR  Adults populations  OR  Adults subjects  OR  Older  OR  Elderly  OR  Elderly people  OR  Older people | Alcohol  OR  Wine  OR  Alcohol consumption  OR  Wine consumption | Migraine  OR  Headache |

**Table S2.** Risk of bias assessment table using Quality Assessment Tool for Observational Cohort and Cross-Sectional Studies.

| **References** |  |  | **Items** | | | | | | | | | | | |
| --- | --- | --- | --- | --- | --- | --- | --- | --- | --- | --- | --- | --- | --- | --- |
|  | **1** | **2** | **3** | **4** | **5** | **6** | **7** | **8** | **9** | **10** | **11** | **12** | **13** | **14** |
| Peatfield et al. 1995 | Y | Y | Y | Y | N | N | N | NR | Y | N | Y | NA | NR | Y |
| Takeshima et al. 2004 | Y | Y | Y | Y | NR | N | N | Y | Y | Y | Y | NA | NR | Y |
| Aamoot et al. 2006 | Y | Y | Y | Y | Y | N | N | Y | Y | N | Y | NA | Y | Y |
| Rist et al. 2016 | Y | Y | Y | Y | Y | N | N | N | Y | N | Y | NA | Y | Y |
| García-Azorín et al. 2020 | Y | Y | Y | Y | N | N | N | N | Y | N | Y | NA | Y | Y |

1.Was the research question or objective in this paper clearly stated?; 2. Was the study population clearly specified and defined?; 3. Was the participation rate of eligible persons at least 50%?; 4. Were all the subjects selected or recruited from the same or similar populations (including the same time period)? Were inclusion and exclusion criteria for being in the study prespecified and applied uniformly to all participants?; 5. Was a sample size justification, power description, or variance and effect estimates provided?; 6. For the analyses in this paper, were the exposure(s) of interest measured prior to the outcome(s) being measured?; 7. Was the timeframe sufficient so that one could reasonably expect to see an association between exposure and outcome if it existed?; 8. For exposures that can vary in amount or level, did the study examine different levels of the exposure as related to the outcome (e.g., categories of exposure, or exposure measured as continuous variable)?; 9. Were the exposure measures (independent variables) clearly defined, valid, reliable, and implemented consistently across all study participants?; 10. Was the exposure(s) assessed more than once over time?; 11. Were the outcome measures (dependent variables) clearly defined, valid, reliable, and implemented consistently across all study participants?; 12. Were the outcome assessors blinded to the exposure status of participants?; 13. Was loss to follow-up after baseline 20% or less?; 14. Were key potential confounding variables measured and adjusted statistically for their impact on the relationship between exposure(s) and outcome(s)?; N: no; NR: not reported; Y: yes.

**Table S3.** Sensitivity analysis.

| **Reference** | **OR** | **LL** | **UL** |
| --- | --- | --- | --- |
| Peatfield et al. 1995 | 1.072 | 0.701 | 1.442 |
| Aamoot et al. 2006 | 0.873 | -0.030 | 1.775 |
| Rist et al. 2016 | 0.688 | 0.110 | 1.265 |
| García-Azorín et al. 2020 | 0.712 | 0.092 | 1.333 |
